# Crop-GPA: an integrated platform of crop gene-phenotype associations

**DOI:** 10.1038/s41540-024-00343-7

**Published:** 2024-02-12

**Authors:** Yujia Gao, Qian Zhou, Jiaxin Luo, Chuan Xia, Youhua Zhang, Zhenyu Yue

**Affiliations:** School of Information and Artificial Intelligence, Anhui Beidou Precision Agriculture Information Engineering Research Center, Anhui Agricultural University, Hefei, Anhui 230036 China

**Keywords:** Computational biology and bioinformatics, Plant sciences

## Abstract

With the increasing availability of large-scale biology data in crop plants, there is an urgent demand for a versatile platform that fully mines and utilizes the data for modern molecular breeding. We present Crop-GPA (https://crop-gpa.aielab.net), a comprehensive and functional open-source platform for crop gene-phenotype association data. The current Crop-GPA provides well-curated information on genes, phenotypes, and their associations (GPAs) to researchers through an intuitive interface, dynamic graphical visualizations, and efficient online tools. Two computational tools, GPA-BERT and GPA-GCN, are specifically developed and integrated into Crop-GPA, facilitating the automatic extraction of gene-phenotype associations from bio-crop literature and predicting unknown relations based on known associations. Through usage examples, we demonstrate how our platform enables the exploration of complex correlations between genes and phenotypes in crop plants. In summary, Crop-GPA serves as a valuable multi-functional resource, empowering the crop research community to gain deeper insights into the biological mechanisms of interest.

## Introduction

Crops are particularly vulnerable to climate change and environmental factors^1,2^. Advancements in crop phenomics research have yielded a vast volume of valuable biological data contributing to improve crop productivity. Over the past decade, gene-phenotype associations (GPAs) data play a crucial role in dissecting the genetic underpinnings of complex traits and guiding the selection of molecular breeding strategies^3–5^. Elucidating the genetic basis of crop breeding requires linking effector genes to multiple phenotypes. Numerous studies have focused on methods that mined the associations between traits and effector genes^4,6,7^. However, challenges such as gene pleiotropy and diverse phenotypic descriptions handicaps the advancements. Emphasizing multidimensional presentation of gene data and phenotype data aids the prediction and exploration of GPAs data. This contributes significantly to the advancement of precise phenotyping strategies in crop breeding. The development of a comprehensive platform for crop GPAs will advance the efficient use of this valuable data.

Genomics and phenomics are stepping into an era of big biological data, marked by significant progress through genome programs and next-generation sequencing technology^4,8^. Despite relevant ontology-driven research, the rapid accumulation of data from public databases and scientific literature presents challenges in integrating, navigating, and analyzing heterogeneous data due to the lack of global standardization. Benefit from the emergence of large-scale gene-disease association datasets^9–11^, significant progress has been achieved in assessing causal relationships between human complex traits and diseases. This includes identifying disease susceptibility genes and biological pathways, interpreting trait-related genetic variants, understanding disease etiology, and developing comprehensive treatment strategies^12–14^. Likewise, the big data of GPAs is also invaluable for crop studies in genomics and phenomics. Despite advancements in genetics identifying GPAs in model species like animals^15^, humans^16^, Drosophila^17^ and Arabidopsis^18^, there are still some defects in previous studies. The task of identifying GPAs from extensive biological resources remains a challenge in crop phenomics. To date, there is no comprehensive platform specifically dedicated to providing GPA data for crops. Standardized GPAs data of rice, maize or wheat are still lacking from most resources, hampering the usability of knowledge about the mechanisms behind biodiversity and adaptation. Understanding inherent relationships of gene and phenotype is vital for breeding new cultivars. Unfortunately, there are currently no databases dedicated to providing clean and abundant GPA data of crops.

Rich resources targeting key agronomic traits didn’t support complex analysis involved in precise crop breeding^3,19,20^, such as plant meta-phenomics database^21^, Plant Trait database TRY^22^, and plant image analysis database^23^. Several genome databases, such as TropGeneDB^24^, AtMAD^25^, PGP^26^, SesameFG^27^, GWAS Atlas^28^, and MaizeGDB^29^, have integrated and utilized genomic, genetic, and phenotypic data. However, these databases have not considered all the necessary factors for crop breeding. The progress commonly relies on manual labeling for data population and updating, which can be costly and time-consuming. However, advancements in artificial intelligence brought an unparalleled opportunity to mine big biological data. Biological relation extraction shifted the process of knowledge discovery to machines by developing computational methods that can automatically mine meaningful facts from vast scientific literature. Deep learning technologies combined with natural language processing (NLP) methods are now widely applied to advance automated entity recognition and relationship mining, such as protein-protein interactions^30^, gene-disease associations^11^, and drug-drug interactions^31^. Few studies have developed models for identifying gene entities and extracting relationships among numerous biological entities, with the aim of extracting GPAs in different species. Trait entity recognition and effective extraction of GPAs from literature remain challenging, especially for crop plants^32^. A versatile machine learning-based pipeline was proposed to automatically identify new relationships in *Arabidopsis* from the body of literature^33^. Recently, several models utilizing pre-trained corpora have been proposed, demonstrating significant performance improvements over Bidirectional Encoder Representation from Transformers (BERT) and other strong baseline models^34,35^. The complexity of crop trait descriptions poses a challenge in accurately identifying trait entities in text. Developing general GPA extraction tools for crops will significantly enhance our ability to extract valuable data.

Modern phenotyping research requires accurate and precise strategies for crop genetic improvements and breeding programs.The identification of crop GPAs will facilitate a better elucidation of whole-organism traits. However, our current understanding of complex GPAs in crops remains limited. Phenomic research incorporating genomics has deciphered the functions of numerous unknown genes and enhanced our understanding of the complex GPAs in various species. Qualitative methods have been studied to predict GPAs for humans and model animals, but very little about crops. These studies will provide testable candidate genes for a wide array of traits in various species. For example, a network-based machine learning model, named diseaseQUEST, is proposed to predict candidate human disease genes for 25 different human diseases and traits^36^. In crop domains, trait-assisted prediction models for crops have been proposed^37,38^. However, as far as we know, more precise computational methods to infer potential GPAs is urgent in crops.

To address the increasing demand, we introduced Crop-GPA, an integrated platform utilizing two computational tools for automated GPAs acquisition, storage, display, and prediction. All the curated gene and phenotypic data have been meticulously organized and represented, serving as a comprehensive repository for mapping complex GPAs. Additionally, a text mining tool based on BERT, named GPA-BERT, was proposed to extract GPAs from literature. We further extend the graph convolutional network (GCN) method to the heterogeneous graph and named it GPA-GCN for predicting potential GPAs. Given the context of phenomics entering the big-data era, our platform serves as a comprehensive repository of information on GPAs, exhibiting multi-function, multi-dimensional, and trans-species characteristics. Crop-GPA is designed as a versatile platform for various research purposes, encompassing the investigation of molecular underpinnings of specific crop traits, and the development and validation of bioinformatics approaches based on gene and phenotypic data.

## Results

### Platform overview

Crop-GPA provides a comprehensive framework for fully describing the genetic and functional mechanisms of crop traits, incorporating associations between genes and phenotypes, along with visualizing their relationship distribution. Crop-GPA is the pioneering dynamic, multi-functional, and scalable platform for crop gene-phenotype associations. It collected 1805 trait entries, 80,676 gene entries, and 374,224 verified GPA entries, covering ten crop species (Table 1). All data are organized in a hierarchical structure. Among them, the trait data for rice is categorized into two groups: the Trait (T) group and the Experiment condition (E) group. Specially, our comprehensive platform offers an intuitive landscape of crop traits, allowing users to browse data from both the gene and phenotype perspectives, while also providing access to specific detailed information. Moreover, two computational tools are integrated to expand the data set and make valuable predictions. As an important data resource in crop research, our platform will be freely accessible and hold significant value for future advancements in genetic engineering, functional genomics, and breeding development in crop plants.

**Table 1:** Summary of the gene-phenotype associations in the Crop-GPA platform

Taxon and Group		Trait	Gene	GPA
Rice (Oryza sativa)	T	339	1269	2197
	E	53	17480	44267
Soybean (Glycine max)		19	30	1205
Alfalfa (Medicago truncatula)		40	31	65
Tomato (Solanum lycopersicum)		72	52	123
Corn (Zea mays)		112	113	262
wheat (Triticum aestivum)		508	5416	131088
Rape (Brassica napus)		30	36356	143818
Cotton (Gossypium hirsutum)		25	6114	18107
Sorghum (Sorghum bicolor)		159	12700	29900
Millet (Setaria italica)		448	1115	3192
Total		1805	80676	374224

T and E represent two categories of the trait data for rice: the Trait (T) group and the Experiment (E) group.

**Fig. 1:**
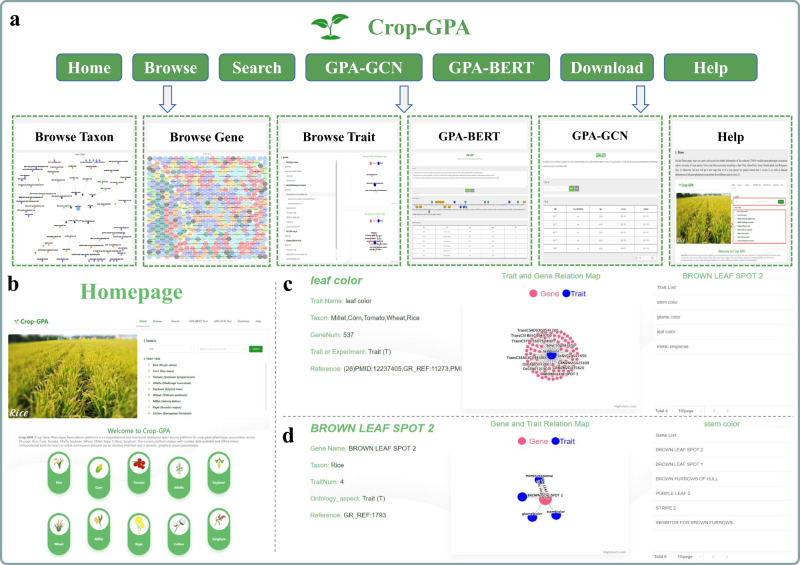
Overview of the Crop-GPA platform. **a** Platform functions include browsing for taxon, gene and trait, searching for genes/traits, tools, download and help. **b** Homepage of Crop-GPA platform. **c** The Gene viewer page. **d** The Trait viewer page. Various interactive plots and charts are also developed for data visualization.

Users can easily navigate the entire database using the menu on the homepage, which includes common tasks such as Home, Browse, Search, GPA-BERT, GPA-GCN, Download, and Document (Fig. 1a). The browsing and retrieval capabilities offered by the platform allow users to access trait data through three different accessions: genes, traits, and the associations. Crop-GPA presents all data through dynamic graphic interfaces and charts, providing an interactive and visually appealing experience for users. Moreover, with the integration of advanced deep learning techniques, two innovative computational tools were developed, named GPA-BERT and GPA-GCN respectively. GPA-BERT functions as a text-mining tool for extracting GPAs, while GPA-GCN serves as a predictor to identify potential relationships between genes and phenotypes using graph convolutional networks and known gene-phenotype association networks. Crop-GPA provides external links with multi crops resources for the extensions of information, including Pubmed^39^, Ontobee (https://ontobee.org/)^40^, GRAMENE (https://archive.gramene.org/)^41^, Ensembl plants^42^, GWAS atlas^28^, Legume Information System^43^, Sorghum QTL Atlas^44^, MDSi^45^ and Sol Genomics Network (SGN)^46^.

#### Search page

A direct search and fuzzy query for species, trait, gene or gene symbol, and the reference can be performed in the search options of Crop-GPA. The Search results page presents a comprehensive list of relationships between traits and their associated genes, including specific phenotypes, involved species, gene symbols, gene names, parent phenotypes, trait groups, and reference IDs. After choosing the phenotype, gene or reference ID of interest in the output form, the results will skip to the details pages including Gene browse page, Trait browse page, and the literature source page on external websites. The list of references provides the corresponding PMID numbers, DOI numbers, and web addresses, all of which are hyperlinked for easy access to the referenced literature.

#### Browse page

##### Taxon browse page

The Species browser provides an easy visualization of information to gain a quick overall understanding of crop trait objects, which is a dynamic web interface. We constructed a family trait tree for each crop species. In the tree diagram representing the trait groups being displayed, a scannable view including multiple heading levels and different colors is organized on the top of the page to better distinguish different trait groups. As shown in Fig. 1a, each trait node (if it has child trait nodes) can be left-clicked, triggering the appearance of a window containing a subgraph and a list that intuitively illustrates the child traits and related genes of the selected trait node. Due to space limitation, only trait data from the root to 4-level for each crop are plotted. Meanwhile, the query function via gene or phenotype is provided so that users can drill down to access complete detailed data.

##### Gene browse page

We allow users to search and browse by gene name or gene symbol (Fig. 1a). The Gene browser gives a graphical overview of phenotype-related genes for ten crops, which is a dynamic visualization of gene data. The genes of different crops are shown in different colors. The pop-up window will display the information of species and gene name when the mouse moves over the gene object. To learn more details about a gene of interest, click on the gene object and go to the specific gene viewer page (Fig. 1c). The gene viewer page starts with the basic gene information, including full gene name, taxon, number of related traits, ontology_aspect and reference source. Specially, the page presents a dynamic visual presentation for the associations between the gene and all its related traits to show the overall relation distributions. Notably, clicking on the gene-related trait node in the association network (Fig. 1c) will display a list of genes directly linked with the selected trait on the right. The detailed information results of gene-related traits are subsequently listed below.

##### Trait browse page

Our platform helps users gain a comprehensive overview of each trait (Fig. 1a). On the trait browse page, the grouped directories of 5 crops are displayed, and two relation maps of the current trait are shown on the right: a map of the trait with its child traits and a map of the trait with its related genes. Users can click the trait object and then go to the trait viewer page (Fig. 1d). The trait viewer page includes four display items: a presentation of basic information about a trait (include trait name, species, number of related genes, ontology group and reference), a dynamic visual presentation of associations between the trait and its related genes, a list of correlated traits associated with the original-trait-related genes, and the detailed information of the related genes from a search in the Crop-GPA. Specifically, a dynamic relation map is presented, including genes or gene symbols that are significantly associated with the query trait.

##### Download and help page

The download page offers users the option to obtain all relevant gene and trait data materials. Additionally, a summary of data collection, processing, and integration is accessible on the help website (https://crop-gpa.aielab.net/help). There, users can find user guides and tutorials on how to navigate the browse, search, and tools functions, along with explanations for each page.

#### GPA-BERT

In addition to the general functions, we developed a text-mining tool to facilitate data integrity maintenance and keep up with updates conveniently. The tool GPA-BERT represents the ability to apply text mining techniques that extracts GPAs from the literature automatically, which helps deepen our current understanding of gene-phenotype interactions in a computable form. The development of GPA-BERT will leverage the knowledge embedded in the enormous amount of scientific literature and facilitate more efficient biocuration of phenotypic data into structured databases.

GPA-BERT is a text analysis tool that utilizes a pre-trained corpus. Users can annotate gene names and trait descriptions in the provided text, allowing for the extraction of gene-phenotype associations based on co-occurrences within the text snippet. On the GPA-BERT page, a text box is provided for users to enter the text, and by clicking the ‘recognize’ button, the tool performs the relation extraction task. The extraction results, representing GPAs, are displayed at the bottom of the page and are manually added to the database after expert verification.

#### GPA-GCN

We developed a computational tool called GPA-GCN within the platform, which is designed to infer potential relationships between genes and phenotypes. On the GPA-GCN page, three functions are provided, including Submit task, Task list, and Search task report. Users first fill in the name and email information, then execute prediction tasks by submitting the required description file of genes or traits after selecting the task type, which gives the gene names or trait terms and their full detailed description. We assign a TaskID for each prediction. The task list shows details of the submitted prediction needs, such as task status (running or completed), creation time and update time, etc. Finally, users can receive the results via email, and the result report will include comprehensive details about the prediction outcomes. The association probabilities of genes and phenotypes could be intuitively illustrated by the calculated metrics. Users can access the result report by email or by searching TaskID on the website.

### Experiments and performance

#### Performance of GPA-BERT

We adopted unsupervised learning approaches to identify bio-crop entities from sentences or text snippets of publications and extract the GPAs. Finally, we obtained 1804 gene entities, 1818 phenotype entities and 10 race entities, resulting in a corpus of GPAs consisting of 672 positive samples and 711 negative samples, which were used to train our BERT-based model. In the two representative bio-crop text mining tasks, our model achieved precision value of 0.633, recall value of 0.692, and F1-measure of 0.659 on named entity recognition. Additionally, for relation extraction, it obtained precision value of 0.613, recall value of 0.683, and F1 score of 0.629. The analysis results compare favorably to the previous state of the art, fully demonstrating that our BERT-based method excels in automatically revealing new GPAs of crops, which were previously not covered by existing relevant resources. Indeed, with the increasing availability of bio-crop literature, our proposed tool can be utilized not only for extracting relationships in the crops studied in this research but also for a diverse range of other crop plants.

#### Implementation and performance of GPA-GCN

Predicting GPAs is essential in functional genomics, especially for crop complex traits influenced by multiple interacting genes. GPA prediction facilitates gene-level understanding of crop behavior and identifies novel target genes for various traits, advancing our knowledge of crop genetics and phenomics. We developed a deep learning-based tool called GPA-GCN (Crop Gene-Phenotype Associations based on Graph Convolutional Network) by integrating Plant Ontology annotations and our GPA database. GPA-GCN is designed for two prediction tasks: predicting gene-related traits (Task-trait) and identifying potential trait-related genes (Task-gene).

##### Effect of k-NN algorithm

**Fig. 2:**
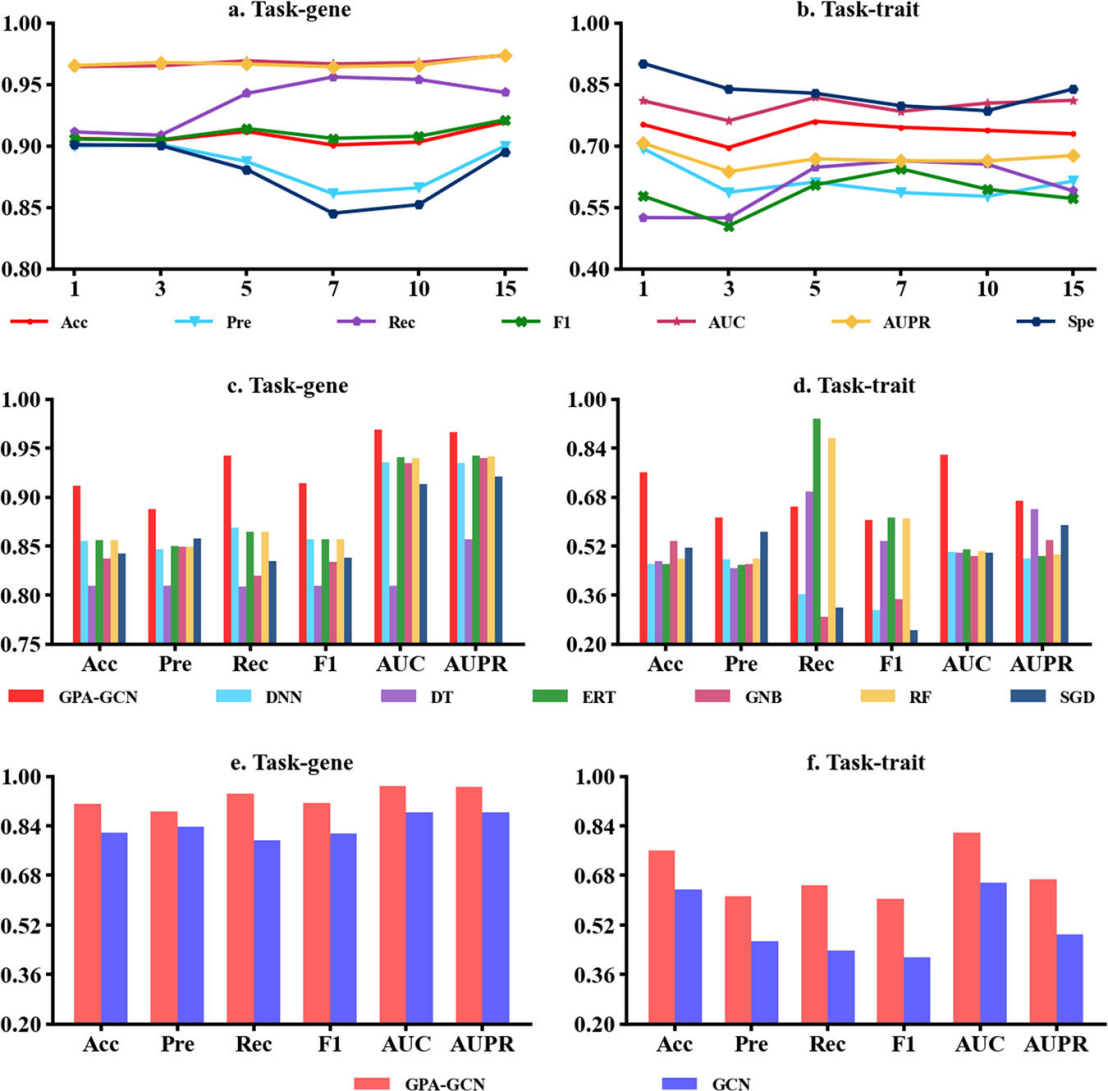
Comparative analysis of prediction performance between GPA-GCN and others. **a**, **b** The effect of k-NN for the prediction performance of GPA-GCN on two tasks, where the abscissa axis represents the number of neighbors. **c**, **d** The comparison of GPA-GCN with the classic machine learning methods on two tasks, including DNN (deep learning-based deep neural network), DT (decision tree), ERT (extremely randomized tree), GNBs (Gaussian naïve Bayes), RF (Random forest), and SGD (Stochastic Gradient Descent). **e**, **f** The comparison of GPA-GCN with the traditional GCN method on two tasks. Acc, Pre, Rec, F1, AUC, and AUPR represent the accuracy, precision, recall, F1-score, the area under the receiver operating characteristic curve, and the area under the precision-recall curve, respectively.

Our proposed tool is developed based on the GCN framework by graph sampling with the feature and topology graph. The k-Nearest Neighbor (k-NN) algorithm is used to construct a full graph based on node features and network topology. For each target node, the k most similar nodes are selected as its neighbors in the graph. The existing studies identify the robustness of the k-NN algorithm with different *k* values for the GCN-based prediction tasks^47^. We conducted experiments to assess the impact of the k-NN algorithm on GPA prediction. The results showed that the prediction performance remained consistent across different values of k (*k* = 1, 3, 5, 7, 10, 15) (Fig. 2a, b), indicating that GPA-GCN is relatively insensitive to the choice of k. The optimal value of k is selected as 5.

##### GPA Prediction comparison

As depicted in Fig. 2c, d we conduct the comparison between GPA-GCN with six classic machine learning algorithms on balanced datasets, including deep learning-based deep neural network (DNN), random forest (RF), extremely randomized trees (ERTs), decision trees (DTs), Gaussian naive Bayes (GNBs), and stochastic gradient descent (SGD). Figure 2e, f shows the comparison between GPA-GCN with the traditional GCN algorithm, and the detailed results on two prediction tasks can be found in the Supplementary Table 1. All experiment conditions are the same as GPA-GCN, including 5-fold cross-validation, random seeds, dataset splitting, topology graph construction, etc. The result shows that GPA-GCN has demonstrated its superiority on two prediction tasks. As shown in Fig. 2d, the recall of GPA-GCN for Task-trait is lower than the others, but the F1 score is worthy of attention and shows a satisfactory performance. Moreover, the result of AUC and AUPR further proves this, reaching 0.969 and 0.967, 0.818 and 0.669, respectively. In addition, a Friedman test is also conducted to prove the performance of the proposed tool outperforms six classic machine learning algorithms. This test confirmed the superiority of GPA-GCN in predicting GPAs.

### Usage examples

Crop-GPA offers users interactive access to genes, traits, and GPA data through its platform’s function module. This enables researchers to explore and analyze the data in an intuitive and user-friendly manner. To showcase the data mining capability of our platform in revealing the intricate relationships between genes and phenotypes, we present usage examples in this section.

**Fig. 3:**
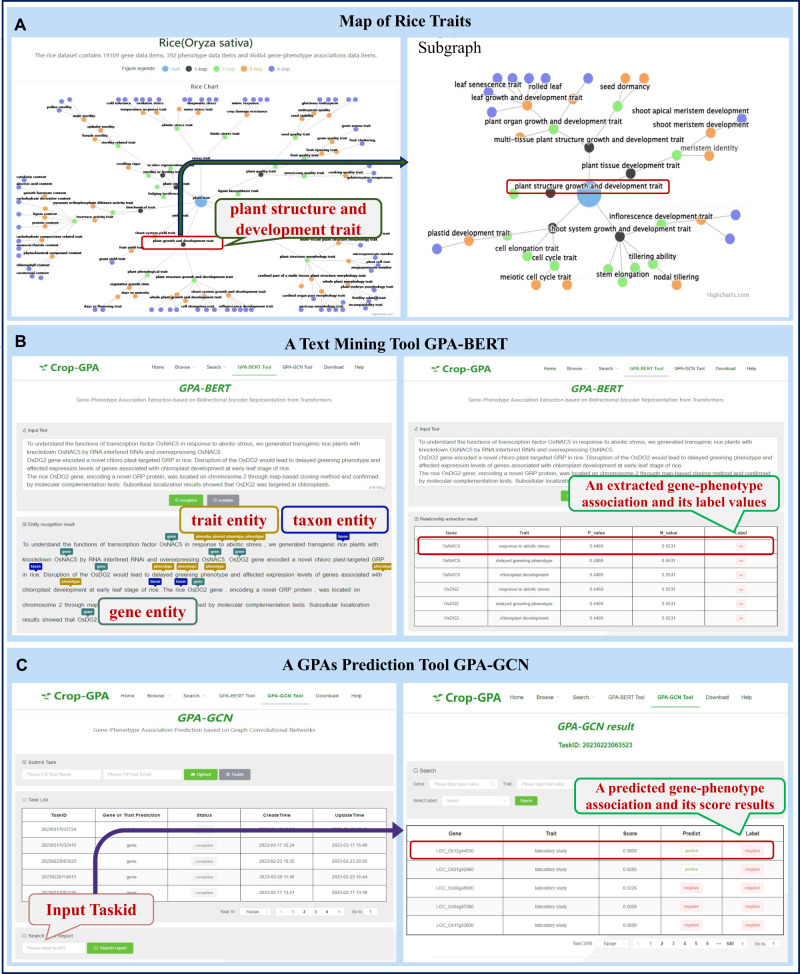
Snapshot views of the usage examples of Crop-GPA. **A** A map of the rice traits, taking the trait “plant structure growth and development trait” as an example. **B** A case of relationship extraction using the GAP-BERT tool, including a text input box, outputs for entity recognition, and results for relationship extraction. **C** An example of predicting GPAs by GPA-GCN, in which “Score” represents the prediction result, “Predict” means the prediction label of a GPA based on a threshold of 0.5, “Label” represents the original label of a GPA if it is present in the dataset.

The gene-phenotype mapping of ten crop species was constructed, and it is displayed as a tree structure for each species, illustrating the dependencies of each trait group (Fig. 3A). Previous studies have shown that complex traits of crops are affected by multiple genes^48^. When clicking on a trait node, a subgraph of the current trait is presented. Specifically, this design of data visualization in Crop-GPA motivates researchers to explore in-depth information about the relationships between genes and phenotypes. Users can access different species regions from the homepage and explore data presentation through links to the Species browse page, Trait browse page, and Gene browse page. This facilitates obtaining the linkage data sets of genes and traits of interest. As an example, we chose rice for our analysis. The current rice dataset contains 46464 GPA entries covering 392 traits connected to 18,749 genes. The results obtained from the platform showed that the “*5-methyltryptophan exposure*” trait, the “*abscisic acid exposure*” trait, and the “*benzothiadiazole exposure*” trait are associated with 156 genes, 1764 genes, and 75 genes, respectively. By analyzing the data, we found four shared genes that are associated with all three traits: LOC_Os01g66120, LOC_Os12g17600, LOC_Os11g07020, and LOC_Os09g30350.

The text mining tool, GPA-BERT, offers a convenient means to keep track of updates to GPA data in the literature, helping researchers stay current with the latest information. Figure 3B shows an example of named entity recognition and relations extraction for GPAs. First, users input a section of literature, such as an abstract or title, into the editable text-input box. Afterward, they click the “recognize” button. In the entity recognition result window, GPA-BERT automatically highlights species entities in blue, gene entities in dark green, and phenotype entities in yellow. Finally, the relation extraction result lists detailed information, including gene name, trait definition, *P*-value, *N*-value, and Label. In Fig. 3B, the first row of the table shows an extracted association between the “OsNAC5” gene and the “response to abiotic stress” trait. The *P*-value of 0.998 indicates the score of the association as a positive sample, while the N_value of 0.002 indicates the score as a negative sample. The Label “yes” indicates the association is a verified entry in our original dataset.

The primary goal of our platform is to collect and curate knowledge on gene data and phenotypic data of crop plants, as well as to extract valuable information from a sufficient number of experimentally validated GPAs data. Users can access GPA-GCN via the menu on the homepage. As illustrated in Fig. 3C, after filling in the name and email, a pop-up window will present and provide a choice of the prediction task type. With the guide of “download example” provided, you will need a file that describes the function of genes or traits based on standard Gene Ontology (GO)^49^, Phenotype And Trait Ontology (PATO), or Plant Experimental Conditions Ontology (PECO). We provided a sample file that contains two sheets and gives basic information about 100 genes entries and 1 trait entry, respectively. In detail, the trait entry is described as “sodium chloride exposure” with the following definition: “A salt exposure (PECO: 0007185) involving the use of sodium chloride salt as a supplement to liquid and soil growth media to study various types of responses on its application.” In Fig. 3C, the prediction task results are presented, and the fields include gene name, trait definition, Score, Predict value, and Label. The field “Score” represents the predicted probability value of a GPA. The field “Predict” and “Label” represent the predicted label and original label of a GPA, respectively.

Together, Crop-GPA offers robust genetic evidence and tools for systematically deciphering the association mechanisms between genes and traits. The functionality enables visual representation of the relationship between genes and phenotypes, facilitating data integration for further interpretation to enhance breeding efforts.

## Discussion

Crop-GPA offers a user-friendly, versatile, and scalable platform for accessing abundant biological data on crop plants. It provides valuable knowledge on species, genes, traits, and gene-phenotype associations, essential for advancing functional genomics and crop breeding. With its comprehensive data and intuitive interface, Crop-GPA empowers researchers to explore and analyze crop research efficiently and effectively. All data on the platform is web-accessible and free to share with the crop community. Notably, we developed advanced tools using NLP technology and deep learning method for keenly updating and predicting more reasonable GPAs. The collaboration of the two proposed tools in Crop-GPA allows for continuous data updates and broader application across various crop plants, ensuring researchers have access to up-to-date and comprehensive information.

Most of the similar previous studies mainly focus on data integrity and diversity, Crop-GPA stands out by integrating gene data, phenotype data and gene-phenotype association data across important crops. This comprehensive approach complements current research efforts effectively. Regarding the way of data organization and presentation, a tree structure is adopted to make it clear for researchers to systematically explore the genetic signals of complex relationships between traits and genes. In addition to dynamically presenting gene-phenotype associations through the platform’s functions, Crop-GPA also offers robust genetic evidence for further data analysis. Taking the trait dataset of rice as an example, there are 339 entries in the Trait (T) group and 53 entries in the Experiment condition (E) group, with 20 pieces of data intersection. Furthermore, several traits are shared across multiple crops. For instance, traits such as “vascular leaf morphology,” “enzyme activity,” and “flowering time” are found in Soybean, Alfalfa, Corn, and Rice. Additionally, our platform enables users to identify significant genes associated with specific traits. In the case of rice, four genes (LOC_Os01g66120, LOC_Os12g17600, LOC_Os11g07020, and LOC_Os09g30350) are associated with all three traits, “5-methyltryptophan exposure”, “abscisic acid exposure”, and “benzothiadiazole exposure”.

As the bio-crop literature continues to grow, applying existing NLP methodologies to crop text mining has limitations. Improved entity recognition and relationship extraction methods are needed in specific fields. Our study proposes an automatic tool based on pre-trained BERT for GPA extraction from texts, enhancing knowledge discovery efficiency. However, challenges persist in systematically describing crop phenotype data with Crop Ontology. Advancements are necessary to comprehensively represent crop phenotype data in our platform. The proposed GPA-BERT can effectively recognize longer crop phenotyping named entities by accurately identifying the exact boundaries of trait descriptions. The results demonstrate that our pre-trained BERT model is capable of identifying complex GPAs from bio-crop or bio-plant texts (Supplementary Table 2). However, it is important to acknowledge that the current GPA-BERT may still make mistakes in recognizing gene and phenotype entities due to certain challenges. These challenges include ambiguous gene symbols of crop plants and the varied descriptions of crop traits in widely shared context patterns. Notably, the analysis of errors in automated GPAs extraction reveals that false positives mainly result from incomplete, incorrect, or unclear gene and non-phrase forms of unfixed-length phenotypic descriptions, which are challenging to exclude during the manual labeling phase. For instance, the word “semidwarf” in the sentence “*The semidwarf phenotype was controlled by the semidwarf gene, sdg*” cannot be accurately labeled as a gene entity or a trait entity^50^. Additionally, some gene symbols are ambiguous, as they may include the name of the species and traits, like “O.sativa homeobox 1”, “Oryza sativa homeobox 1”, “Dwarf 1”, and “dwarf 89”^51–53^. Furthermore, certain words often appear in both gene and trait entities, such as ‘culm’^54^. To enhance the performance of GPA extraction, future studies can focus on more detailed division of trait descriptions and relation boundaries.

In recent years, crop phenomics studies have highlighted the importance of establishing gene-phenotype correlations in modern crop genetics, aiding in the identification of trait-related genes for crop breeding. However, existing methods for GPAs primarily focus on humans and mammals, and their accuracy and reliability for crops need improvement. To address these limitations, we propose a specialized method based on a GCN-based framework that integrates gene semantic similarity and trait semantic similarity from known GPAs. Our GPA-GCN model adaptively extracts correlated information from node features and topology graphs, achieving outstanding performance with an AUC of 0.969 and an AUPR of 0.966 in 5-fold cross-validation, surpassing existing machine learning and classic GCN methods. GPA-GCN’s strength lies in its ability to prioritize important nodes and reduce edge noise, improving performance. Multiple external lines of evidence have demonstrated the predictive capabilities of GPA-GCN, which surpass those of traditional machine learning methods and classical GCN approaches. The model exhibits superior performance on an independent test set (Fig. 2), providing an objective assessment of its accuracy and generalizability. Furthermore, we conducted predictions for three traits in the experimental condition group (E) of rice, including “Magnaporthe grisea exposure”, “calcium carbonate exposure”, and “temperature environment exposure”. The predicted GPAs encompassed a set of genes such as LOC_Os01g70090, LOC_Os09g39560, LOC_Os08g01910, and LOC_Os01g55390, etc, which have been previously reported in the literature^55,56^. The aforementioned case studies have demonstrated the effectiveness of the model in predicting potential GPAs.

In the current Crop-GPA, of the 10,566 genes with at least one phenotype hit, 2964 showcased a distinctive gene-phenotype association by linking with multiple traits. Overall, Crop-GPA offers a comprehensive repository and two computational tools for the analysis and exploration of crop phenotypic studies. The platform provides a convenient way to gain a quick overall understanding of genes and traits, allowing users to intuitively access information about an entire gene-phenotype association data by a visual representation. Moving forward, we will continue to integrate new data from available resources and update our database regularly, mainly for gene and phenotype data from large-scale genomics projects. Our work addresses the crucial challenge of reusing a vast amount of literature data and leveraging it for prediction purposes. This effort lays a solid foundation for enhancing our understanding of crop phenotypic diversity and paves the way for formulating more effective and accurate crop breeding strategies.

## Methods

**Fig. 4:**
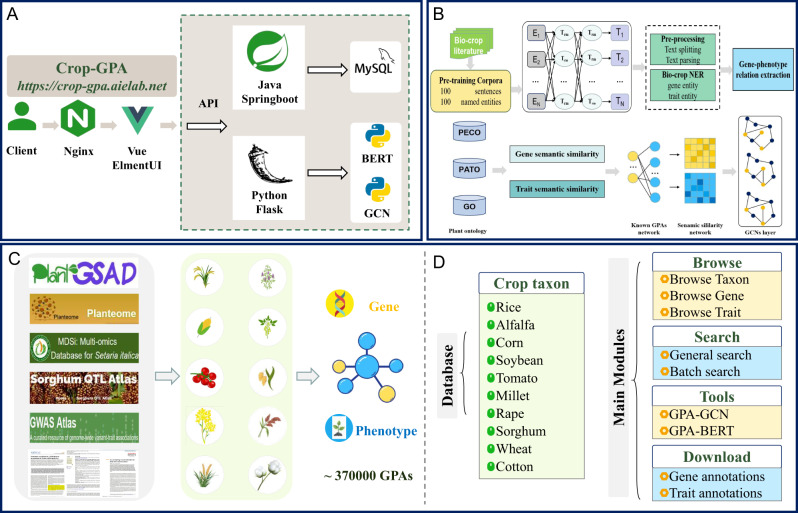
Workflow of the Crop-GPA. **A**, **B** Architecture and principles of the platform. **C** Data materials were manually collected from PlantGSAD, Planteome, MDSi, Sorghum QTL Atlas, GWAS Altlas, and public literature. **D** Composition of the platform.

The workflow of Crop-GPA is illustrated in Fig. 4, summarizing the data sources, platform composition, and its underlying principles.

### Extraction of GPAs based on BERT

To alleviate the burden of manual curation and extract valuable information from literature, we have developed an NLP data processing pipeline. This pipeline enables us to automatically mine potential gene-phenotype linkages from a vast amount of literature, saving time and effort while uncovering valuable insights. Specifically, two modules are developed in our tool GPA-BERT: (1) A Named Entity Recognition (NER) module to identify numerous proper nouns from the biological corpus; (2) A BERT-based Relationship Extraction (RE) module to classify relations between entities. After applying filters to the publication sources, we employ a manual annotation method to annotate gene entities, trait entities, and relation entities in the literature. This manual annotation process ensures the accuracy and reliability of the extracted information from the literature. Taking inspiration from recent research progress on relation extraction^30,57^, we took a pre-trained BERT model on our annotated dataset to prepare a task model for name and relation extraction.

In Fig. 4B, the input text is processed through the pre-trained language representation architecture. GPA-BERT is initialized with BERT weights and then further pre-trained on bio-crop domain corpora containing domain-specific proper nouns and terms. The model is fine-tuned for the NER and RE tasks. Precision, recall, and F1 score are used for evaluating the performance of GPA-BERT.

### Data materials

Data material utilized in this study were collected from diverse resources. The primary crop material consisted of two parts: one part was curated from journal literature through manual collation, and the other part was obtained from public plant databases. The corpus of literature data built in the current is derived from two sources. Initially, trait data materials were gathered from the plant gene set annotation database PlantGSAD^58^ and Planteome^37^. These databases curate gene set annotation data from various public databases and offer reference and species-specific plant ontologies as standardized resources for researchers. Additionally, 1075 full-text PDFs relevant to rice genomics were obtained^59^ and constituted a vital segment of our corpus. Moreover, we retrieved relevant literature from PubMed as supplementary materials, focusing on recent years and filtering the search with keywords such as ‘trait,’ ‘phenotype,’ ‘phenomic,’ and ‘gene,’ among others, across ten crop species: Rice, Corn, Soybean, Tomato, Alfalfa^60^, Wheat, Sorghum, Rape, Cotton, and Millet. Further data sources included the Sorghum QTL Atlas^44^, *Brassica napu*s^61^, Setaria italica^45^, and GWAS Atlas^28^, etc. Finally, we manually processed all the derived data to remove redundancy and conflicts. Through this process, a total of 374224 GPAs were identified and marked based on plant ontology references. To ensure organization and categorization, all entries in Crop-GPA were assigned unique traitIDs and categorized into E and T groups.

### GCN-based prediction

We proposed a computational tool called GPA-GCN to predict new traits (task Tt) and new genes (task Tg) based on known GPAs through GCN with graph sampling technique^47^. The method is mainly composed of two crucial steps:

First, define the node (i.e. genes and phenotypes) feature and construct the topology graph by the *k*-Nearest Neighbors (*k*-NN) algorithm to explore the most relevant and helpful information for GPAs. As shown in the Supplementary Table 2, we crawled and constructed a balanced dataset, involving in 23558 GPA entries, 12187 genes, and 32 traits. With the assumption that the genes with similar functions are more likely to be associated with similar phenotypes, we calculated the semantic similarity based on the descriptions of genes and phenotypes, and obtained two similarity matrixes: gene semantic similarity matrix ${\rm{GSSM}}({g}_{i},{g}_{j})$ and phenotype semantic similarity matrix ${\rm{PSSM}}({p}_{i},{p}_{j})$. The Principle Components Analysis method is adopted to reduce the dimensionality of the GSSM from 11177 to 10. We spliced the GSSM and PSSM by row as the input feature matrix $M$ for the GPAs prediction task. Each row of $M$ represents a gene-phenotype pair ${{GPP}}_{i}$, which is a combined vector consisting of both gene features and phenotype features. The equation of matrix ${\rm{M}}$ is:

1 $${{{M={({GPP}}_{i})}_{47716}=({Vector}}_{{gene}(10)},{{Vector}}_{{phenotype}(32)})}_{(47716* 42)}$$

Each node in the map represents the gene-phenotype pair (GPP) and the node label distinguishes whether the GPP is a GPA or not. The edge in the graph represents similarity about this node and its k nearest neighbors. Thus, we construct a graph $G(V,E,M)$, where $V$ is the set of GPP nodes, $E$ is the set of edges, and $M$ is the node feature matrix. Each node is classified by executing the GCN algorithm on the graph $G$. The GCN method fused the feature of the node itself and the information that is aggregated and updated from a wider range of neighbor nodes and edges.

Second, we apply the graph sampling algorithm to GCN for relation prediction tasks. Specifically, we sample N subgraphs from the original graph G(V, E, M) and construct a complete GCN on each subgraph to perform an unsupervised node classification task. This approach allows us to efficiently process large graphs and extract relevant information for the prediction tasks. ${E}_{s,m}$ is a set of $m$ edges randomly sampled from $E$ according to the optimal probability ${P}_{{e}_{(i,j)}}$ of edge(i, j), ${V}_{s}$ is a set of nodes that are the end-points of edges in ${E}_{s,m}$. The subgraph that consists of nodes ${V}_{s}$ is defined as follows:

2 $${G}_{s,n}=\left({V}_{s},{E}_{s,m}\right),n=1,\ldots ,N$$

where

3 $${P}_{{e}_{(i,j)}}=\frac{\frac{1}{{Deg}(i)}+\frac{1}{{Deg}(j)}}{\sum \left({i}^{{\prime} },{j}^{{\prime} }\right)\in E(\frac{1}{{Deg}({i}^{{\prime} })}+\frac{1}{{Deg}({j}^{{\prime} })})}$$

After the iterative process of graph sampling, we construct GCN on each subgraph ${G}_{s,n}$. The propagation model from the ${l}_{{th}}$ layer to the ${(l+1)}_{{th}}$ layer is as follows:

4 $${H}^{(l+1)}={\rm{\sigma }}\,({\widetilde{D}}^{-\frac{1}{2}}\,\widetilde{A}\,{\widetilde{D}}^{\frac{1}{2}}\,{H}^{\left(l\right)}{W}^{\left(l\right)})$$

where $\widetilde{A}=A+{I}_{N}$, $A$ is the adjacency matrix, ${I}_{N}$ is the identity matrix, $\widetilde{D}$ is the degree matrix of nodes, ${H}^{(l)}$ is the feature of the ${l}_{{th}}$ layer (${H}^{0}=M$), ${W}^{(l)}$ is the weight of the ${l}_{{th}}$ layer, ${\rm{\sigma }}$ is the nonlinear activation function.

### Platform development

Crop-GPA was built on a CentOS server (8.0). All trait data, gene data, and association relations information were organized and stored in the MySQL database (https://www.mysql.com, version 5.7). The platform was integrated into a graphical interface by using HTML5, CSS3, and Vue technologies. In addition, two tools, GPA-BERT and GPA-GCN, were implemented using Python and encapsulated within the Flask framework. Crop-GPA is freely available at https://crop-gpa.aielab.net. Any feedback about Crop-GPA is welcome.

### Reporting summary

Further information on research design is available in the Nature Research Reporting Summary linked to this article.

### Supplementary information

Supplementary Materials

Reporting summary

## Data Availability

The majority of the data used in this study is available for download on Crop-GPA at https://crop-gpa.aielab.net. Raw data is available from the authors upon request.

## Code availability

The codes used in this study are available at: https://github.com/AhauBioinformatics/Crop-GPA.
