# Supplementary material for: Crop-GPA: an integrated platform of crop gene-phenotype associations
Source: NPJ Syst Biol Appl. 2024 Feb 12;10:15. doi: 10.1038/s41540-024-00343-7 (PMC10861494; doi:10.1038/s41540-024-00343-7)
Supplement: Supplementary file 1 — Supplementary Materials [file 41540_2024_343_MOESM1_ESM.pdf]

Crop-GPA: an Integrated Platform of Crop Gene-phenotype Associations

Yujia Gao[a,#], Qian Zhou[a,#], Jiaxin Luo[a,#], Youhua Zhang[a], Chuan Xia[a], and Zhenyu Yue[a,*]
[a]School of Information and Artificial Intelligence, Anhui Provincial Engineering Research Center for Beidou Precision Agriculture Information, Anhui Agricultural University, Hefei, Anhui 230036, China

Supplementary materials

Supplementary Table 1. Comparison results of 5-fold cross-validation for two prediction tasks with six classic machine learning methods.

Method	Task	Accuracy	Precision	Recall	F1-score	AUC	AUPR
GPA-GCN	Tg	0.91177	0.88740	0.94272	0.91422	**0.96925**	**0.96665**
	Tt	0.76008	0.61190	0.64776	0.60480	**0.81804**	**0.66862**
DNN	Tg	0.85508	0.84614	0.86848	0.85702	0.93576	0.93490
	Tt	0.46360	0.47508	0.36494	0.31162	0.50060	0.47824
DT	Tg	0.80936	0.80942	0.80926	0.80930	0.80938	0.85698
	Tt	0.46982	0.44818	0.69772	0.53684	0.49868	0.64176
ERT	Tg	0.85570	0.84972	0.86420	0.85688	0.94038	0.94202
	Tt	0.46214	0.46050	0.93500	0.61206	0.50808	0.48690
GNB	Tg	0.83680	0.84872	0.81972	0.83396	0.93500	0.93956
	Tt	0.53542	0.46352	0.28910	0.34860	0.48568	0.53808
RF	Tg	0.85556	0.84914	0.86468	0.85682	0.94014	0.94182
	Tt	0.47986	0.47914	0.87430	0.61060	0.50394	0.49274
SGD	Tg	0.84200	0.85760	0.83448	0.83822	0.91292	0.92128
	Tt	0.51490	0.56564	0.32034	0.24562	0.49902	0.58970

Note: Tg refers to Task-gene, the prediction task of trait-related genes. Tt refers to Task-trait, the prediction task of gene-related traits. DNN represents the deep neural network. DT represents the decision tree. ERT represents the extremely randomized tree. GNB represents the gaussian naïve baye. RF represents the random forest. SGD represents the stochastic gradient descent.

Supplementary Table 2. Summary of the dataset and prediction performance of GPA-BERT.

Named entity recognition	Named entity			Performance		
	Crop	Gene	Phenotype	F1	Precision	Recall
	10	1804	1818	0.659	0.633	0.692
Relation extraction	Dataset			Performance		
	Positive		Negative	F1	Precision	Recall
	672		711	0.629	0.613	0.683

Supplementary Table 3. Summary of the balanced dataset of GPA-GCN.

Dataset		GPA	Gene	Trait
Balanced	Positive	23558	12187	32
	Negative	23558	12187	32
	Total	47116	12187	32
