# Supplementary material for: Crop-GPA: an integrated platform of crop gene-phenotype associations
Source: NPJ Syst Biol Appl. 2024 Feb 12;10:15. doi: 10.1038/s41540-024-00343-7 (PMC10861494; doi:10.1038/s41540-024-00343-7)
Supplement: Supplementary file 2 — Reporting summary [file 41540_2024_343_MOESM2_ESM.pdf]

nature portfolio

Corresponding author(s): Youhua Zhang and Zhenyu Yue

Last updated by author(s): Nov 18, 2023

Reporting Summary

Nature Portfolio wishes to improve the reproducibility of the work that we publish. This form provides structure for consistency and transparency in reporting. For further information on Nature Portfolio policies, see our Editorial Policies and the Editorial Policy Checklist.

Statistics

For all statistical analyses, confirm that the following items are present in the figure legend, table legend, main text, or Methods section.

n/a	Confirmed	
☐	☒	The exact sample size (*n*) for each experimental group/condition, given as a discrete number and unit of measurement
☒	☐	A statement on whether measurements were taken from distinct samples or whether the same sample was measured repeatedly
☒	☐	The statistical test(s) used AND whether they are one- or two-sided<br>*Only common tests should be described solely by name; describe more complex techniques in the Methods section.*
☒	☐	A description of all covariates tested
☒	☐	A description of any assumptions or corrections, such as tests of normality and adjustment for multiple comparisons
☐	☒	A full description of the statistical parameters including central tendency (e.g. means) or other basic estimates (e.g. regression coefficient) AND variation (e.g. standard deviation) or associated estimates of uncertainty (e.g. confidence intervals)
☒	☐	For null hypothesis testing, the test statistic (e.g. *F*, *t*, *r*) with confidence intervals, effect sizes, degrees of freedom and *P* value noted<br>*Give P values as exact values whenever suitable.*
☒	☐	For Bayesian analysis, information on the choice of priors and Markov chain Monte Carlo settings
☐	☒	For hierarchical and complex designs, identification of the appropriate level for tests and full reporting of outcomes
☒	☐	Estimates of effect sizes (e.g. Cohen's *d*, Pearson's *r*), indicating how they were calculated

Our web collection on statistics for biologists contains articles on many of the points above.

Software and code

Policy information about availability of computer code

Data collection	In addition to downloading data from public datasets, we used Python web scrapers (Python 3.7) for data retrieval through web crawling and extraction. No other software or code was involved in the data collection process.
Data analysis	Our research data analysis and visualization were conducted on a platform built on a CentOS server (version 8.0). All data were organized and stored in the MySQL database (version 5.7, https://www.mysql.com). The platform included a graphical interface developed with HTML5, CSS3, and Vue technologies. Specific analysis tasks were implemented using Python and encapsulated within the Flask framework.

For manuscripts utilizing custom algorithms or software that are central to the research but not yet described in published literature, software must be made available to editors and reviewers. We strongly encourage code deposition in a community repository (e.g. GitHub). See the Nature Portfolio guidelines for submitting code & software for further information.

Data

Policy information about availability of data

All manuscripts must include a data availability statement. This statement should provide the following information, where applicable:

- Accession codes, unique identifiers, or web links for publicly available datasets
- A description of any restrictions on data availability
- For clinical datasets or third party data, please ensure that the statement adheres to our policy

The majority of the data used in this study is available for download on Crop-GPA at https://crop-gpa.aielab.net. Raw data is available from the authors upon request.

Research involving human participants, their data, or biological material

Policy information about studies with human participants or human data. See also policy information about sex, gender (identity/presentation), and sexual orientation and race, ethnicity and racism.

Reporting on sex and gender	Our research does not include the human sex and gender component. These aspects are not within the scope of our study.
Reporting on race, ethnicity, or other socially relevant groupings	Our research does not include the race, ethnicity, or other socially relevant groupings component. These aspects are not within the scope of our study.
Population characteristics	Our research does not include the population component. This aspect is not within the scope of our study.
Recruitment	Our research does not include the recruitment component. This aspect is not within the scope of our study.
Ethics oversight	Our research does not include the ethics oversight component. This aspect is not within the scope of our study.

Note that full information on the approval of the study protocol must also be provided in the manuscript.

Field-specific reporting

Please select the one below that is the best fit for your research. If you are not sure, read the appropriate sections before making your selection.

☒ Life sciences ☐ Behavioural & social sciences ☐ Ecological, evolutionary & environmental sciences

For a reference copy of the document with all sections, see nature.com/documents/nr-reporting-summary-flat.pdf

Life sciences study design

All studies must disclose on these points even when the disclosure is negative.

Sample size	Our study focused on establishing a functional platform of gene-phenotype associations for crops. Data selection involved rigorous screening based on P-value and other indicators, ensuring diversity across species and meeting the requirements for deep learning modeling. The determination of the sample size relied on the availability and diversity of data within our chosen domain. Our objective was to encompass a wide spectrum of instances to bolster the robustness and applicability of the database. This approach aligns with the overarching purpose of the study, which is to provide a comprehensive resource for gene-phenotype associations in crops.
Data exclusions	The data in the study are sourced from publicly available databases or published literature. However, some data were excluded due to incomplete information. Quality assurance for specific GWAS data was achieved through screening based on P-value.
Replication	The findings in the study are both reproducible and verifiable.
Randomization	This question is not applicable to the study. The sample allocation in this study was not random but meticulously designed to meet the training and prediction requirements of deep learning modeling. Given the nature of our study, covariate control was not employed.
Blinding	Blinding was not relevant to our study as the experimental design did not involve subjective assessments that could be influenced by knowledge of group allocation.

Reporting for specific materials, systems and methods

We require information from authors about some types of materials, experimental systems and methods used in many studies. Here, indicate whether each material, system or method listed is relevant to your study. If you are not sure if a list item applies to your research, read the appropriate section before selecting a response.

Materials & experimental systems

n/a	Involved in the study
☒	☐ Antibodies
☒	☐ Eukaryotic cell lines
☒	☐ Palaeontology and archaeology
☒	☐ Animals and other organisms
☒	☐ Clinical data
☒	☐ Dual use research of concern
☐	☒ Plants

Methods

n/a	Involved in the study
☒	☐ ChIP-seq
☒	☐ Flow cytometry
☒	☐ MRI-based neuroimaging

Dual use research of concern

Policy information about dual use research of concern

Hazards

Could the accidental, deliberate or reckless misuse of agents or technologies generated in the work, or the application of information presented in the manuscript, pose a threat to:

No	Yes
☒	☐ Public health
☒	☐ National security
☒	☐ Crops and/or livestock
☒	☐ Ecosystems
☒	☐ Any other significant area

Experiments of concern

Does the work involve any of these experiments of concern:

No	Yes
☒	☐ Demonstrate how to render a vaccine ineffective
☒	☐ Confer resistance to therapeutically useful antibiotics or antiviral agents
☒	☐ Enhance the virulence of a pathogen or render a nonpathogen virulent
☒	☐ Increase transmissibility of a pathogen
☒	☐ Alter the host range of a pathogen
☒	☐ Enable evasion of diagnostic/detection modalities
☒	☐ Enable the weaponization of a biological agent or toxin
☒	☐ Any other potentially harmful combination of experiments and agents

Plants

Seed stocks	The data employed in this study were meticulously curated from diverse sources. The primary crop material comprised two components: one extracted through a comprehensive manual collation of journal literature, and the other sourced from authoritative public plant databases, including PlantGSAD and Planteome. A vital segment of our dataset consisted of 1075 meticulously selected full-text PDFs relevant to rice genomics. Supplementary materials were acquired from PubMed to augment our dataset. Furthermore, our data sources were expanded to encompass the Sorghum QTL Atlas, Brassica napus, Setaria italica, GWAS Atlas, among others.
Novel plant genotypes	Our research focus does not involve the generation of novel plant genotypes through processes such as transgenic approaches, gene editing, chemical/radiation-based mutagenesis, or hybridization.
Authentication	Our research did not involve the use of seed stocks or the generation of novel genotypes□ Our primary focus was on curating gene-phenotype associations data and conducting predictive modeling based on this foundation. Authentication and mutation assessment procedures were not part of the scope of our investigation.
